# Analgesic outcomes of 650 nm versus 810 nm diode laser photobiomodulation after dental implant placement in a randomized controlled trial

**DOI:** 10.1038/s41598-025-32248-8

**Published:** 2026-04-21

**Authors:** Mohamed M. Y. Abdelsalam, Zein Z. A. Adham, Reema Saeed-ur-Rahman, Ahmed Abbas Zaky

**Affiliations:** 1 Department of Medical Applications of LASER, Cairo University, National Institute of Laser Enhanced Sciences (NILES), Giza, Egypt; 2 Faculty of Dentistry, Cairo University, Giza, Egypt; 3 Faculty of Oral and Dental Medicine and Surgery, University of Tripoli, Tripoli, Libya; 4 Gandhara University, Peshawar, Pakistan

**Keywords:** Photobiomodulation, Dental implants, Diode laser, Postoperative pain, 650 nm, 810 nm, Numeric Rating Scale, OHIP-14, OHRQoL, Analgesic use, Clinical trial, Biophysics, Medical research, Signs and symptoms

## Abstract

Photobiomodulation (PBM) is a promising non-pharmacological approach for managing postoperative pain after dental implant placement. Yet, comparative data on red (650 nm) and near-infrared (810 nm) diode lasers remain limited and inconclusive. This double-blind, randomized controlled trial evaluated the short-term analgesic efficacy of 650 nm and 810 nm PBM on postoperative pain, analgesic intake, and oral health-related quality of life (OHRQoL) following single-implant placement in the posterior maxilla. Sixty patients were randomly assigned to receive PBM with 650 nm, PBM with 810 nm, or a sham control. The full treatment protocol involved a total of six laser therapy sessions (18 J total energy per site, three sessions per week for two weeks). However, for the primary outcome of acute pain evaluation, only the first two sessions were included in the analysis (immediately postoperatively and at 48 hours). Pain intensity (11-point Numeric Rating Scale) and analgesic use were recorded at 2, 6, 12, 24, 48, and 72 hours post-surgery. OHRQoL was assessed using the Oral Health Impact Profile-14 (OHIP-14) questionnaire at baseline, one week, and after crown delivery, thereby encompassing the full six-session protocol. At 2 hours, both PBM groups reported significantly lower pain scores compared to the sham group (p < 0.05), with no severe pain observed in the PBM groups. The 650 nm group exhibited significantly reduced analgesic consumption at 2 and 6 hours (p < 0.05), indicating a superior early analgesic effect. Pain scores and medication use converged across groups after Day 1, with no significant differences observed by Day 2 and Day 3, indicating that the analgesic effect was transient and limited to the acute phase. While overall OHRQoL scores did not differ significantly, the 810 nm group showed improvement in the “physical disability” domain. These results suggest that PBM, particularly at 650 nm, may serve as an effective adjunct to improve early postoperative outcomes in minimally invasive implant procedures.

Trial registration: The study is retrospectively registered at ClinicalTrials.gov (https://clinicaltrials.gov/study/NCT06988722 ; Identifier: NCT06988722, registered on 25/05/2025).

## Introduction

Dental implant procedures have revolutionized oral rehabilitation, offering durable solutions for tooth loss. However, postoperative complications such as pain and swelling can impair healing and negatively impact patients’ oral health-related quality of life (OHRQoL). Effective pain management is crucial, as inadequate control can lead to discomfort, delayed recovery, and reduced patient satisfaction^1^.

Photobiomodulation (PBM) therapy, utilizing low-level laser energy, has emerged as a non-invasive method to promote tissue healing and reduce inflammation. The therapeutic effects of PBM are primarily attributed to its interaction with cellular mitochondria^2^. Diode laser photons are absorbed by chromophores, particularly cytochrome c oxidase, within the mitochondrial respiratory chain, stimulating adenosine triphosphate (ATP) production, enhancing cellular energy levels, and accelerating tissue repair^2^. Additionally, PBM triggers the release of endorphins, contributing to immediate postoperative pain relief, and inhibits the cyclooxygenase (COX) pathway, reducing the synthesis of pro-inflammatory prostaglandins^2,3^.

Post-implant surgery pain significantly influences a patient’s OHRQoL, affecting functions such as speaking, chewing, and maintaining oral hygiene. PBM therapy offers a non-pharmacological approach to minimize these issues, thereby enhancing postoperative recovery and overall patient satisfaction. A study by Alzarea (2016) found that patients with dental implants reported satisfaction with their OHRQoL when assessed using the Oral Health Impact Profile (OHIP-14) questionnaire^4^. However, there is a notable lack of data regarding the specific influence of PBM on OHRQoL, highlighting the need for further research to understand its broader impact beyond pain relief.

Diode lasers emitting at 810 nm and 650 nm wavelengths are commonly employed in implant dentistry, each offering distinct therapeutic effects^5^. The 810 nm wavelength, classified as near-infrared light, penetrates deeper into soft tissues and bone structures, facilitating anti-inflammatory action and reducing edema. Conversely, the 650 nm diode laser, within the red light spectrum, primarily affects surface tissues, reducing epithelial irritation and enhancing wound epithelialization^6^.

Although 810 nm and 650 nm diode lasers are widely used, comparative studies specifically evaluating their efficacy in managing postoperative pain following dental implant surgery remain limited^6,7^. Furthermore, existing research on PBM often suffers from methodological limitations, including inconsistent reporting, lack of standardization, and inadequate randomization and blinding, potentially compromising the reliability of the results. Additionally, studies have reported conflicting outcomes regarding PBM’s efficacy, further emphasizing the need for well-designed trials to establish a solid evidence base for its clinical application^1^. A systematic review by Rodriguez Salazar et al. (2023) also highlighted the need for standardized PBM protocols to enhance clinical reliability^8^. Similarly, Farazi et al. (2024) discussed the therapeutic potential of PBM across various medical applications but noted inconsistencies in study methodologies^9^.

The maxillary molar region was selected to standardize implant placement and avoid variability, as implant site and proximity to vital structures can influence postoperative pain and stability^10^. This site is among the most frequently lost tooth positions^11^, often presenting with low bone density^12^ and anatomical constraints such as sinus proximity^13^, making it clinically relevant for evaluating treatment efficacy.

This randomized controlled trial aims to compare the efficacy of 810 nm and 650 nm diode lasers in alleviating pain after dental implant surgery. The primary objective is to evaluate the impact of PBM on pain management by assessing pain levels using the Numerical Rating Scale (NRS). As secondary objectives, the study will investigate the influence of PBM on analgesic consumption and OHRQoL using the OHIP-14 questionnaire. Furthermore, the study will explore potential correlations between pain management, analgesic intake, and OHRQoL outcomes to provide a comprehensive understanding of PBM’s therapeutic effects.

We hypothesize that PBM with both wavelengths will effectively alleviate postoperative pain, diminish the reliance on analgesic intake, and improve OHRQoL, with potential differences in efficacy between the two wavelengths. By implementing rigorous study design protocols, including proper blinding and randomization, this research seeks to provide high-quality evidence on the optimal PBM wavelength for pain management in dental implant patients.

## Methods

### Study design

This randomized, controlled, parallel-group clinical trial was conducted at the National Institute of Laser Enhanced Sciences (NILES), Cairo University, between August 2023 and June 2024. The study adhered to CONSORT guidelines, and all methods were carried out following relevant guidelines and regulations in compliance with the Declaration of Helsinki. It forms part of the first author’s broader PhD project, which received ethical approval from the **NILES Ethics Committee (Approval No. NILES-EC-CU 23/7/18(In)).** Written informed consent was obtained from all participants, and a copy of the consent form template is provided in Supplementary File 1. The study is *retrospectively* registered at **ClinicalTrials.gov** **(** https://clinicaltrials.gov/study/NCT06988722**; Identifier: NCT06988722, registered on 25/05/2025).** No changes were made to the trial protocol or the prespecified outcomes after the start of the trial.

#### Sample size calculation

The sample size was calculated using G*Power software (Version 3.1.9.7, Franz Faul, Universität Kiel, Germany), accessed in June 2023, to achieve 80% power at a significance level $\alpha$ of 0.05. A clinically significant difference of 2 points on the Numerical Pain Rating Scale (NRS) was anticipated, based on Farrar et al. (2001)^14^, and a standard deviation of 2.9 for NRS was conservatively assumed, informed by values reported in Genc Sen and Kaya (2019)^15^, Safdari et al. (2018)^16^, Koparal et al. (2018)^17^, and Bourgault et al. (2015)^18^. Accounting for attrition, the analysis determined a minimum of 20 participants per group.

#### Participants eligibility criteria

Participants were adults aged 20–65 years in good general and oral health, classified as ASA I or II, and indicated for a single implant in a healed posterior maxillary site without need for grafting. Exclusion criteria included recent use of analgesics or corticosteroids, alcohol abuse, chronic pain, systemic diseases, pregnancy or breastfeeding, photosensitivity or contraindications to laser therapy, allergy to acetaminophen, prior surgery at the same site, and heavy smoking (>12 cigarettes/day) or recent cessation within six months.

Patients were recruited during routine clinical visits. Eligibility was explained in detail, and those meeting the inclusion criteria were enrolled in the study.

#### Randomization and blinding

Participants were randomly assigned to three groups (1:1:1) using an online sequence generator (GraphPad Software, Boston, MA, USA, https://www.graphpad.com,) stratified by sex using a fixed block size of six. Allocation was concealed in sequentially numbered, opaque, sealed envelopes prepared by a non-study research assistant, maintaining blinding for participants and data collectors. The surgeon was unblinded, as device operation and parameter adjustment were required; however, performance bias was minimized through a fully standardized, time-controlled protocol. All patients received identical postoperative care and analgesics.

#### Surgical protocol

A standardized surgical protocol was meticulously followed to ensure consistency and minimize confounding factors influencing post-implantation pain. These factors included the operator experience, number of implants, implantation site, inflammation status, sex, age, procedure duration, and implant size, all of which have been shown to affect postoperative pain^10,19^. The surgical procedure was standardized as follows:

1. **Preoperative Evaluation:** Cone-beam computed tomography (CBCT) was performed to confirm sufficient bone volume for implant placement.
2. **Anesthesia:** Local anesthesia (2% lidocaine with 1:100,000 epinephrine) was administered.
3. **Flap Design:** A minimally invasive H-shaped, full-thickness flap was raised to preserve adjacent papillae and reduce surgical trauma.
4. **Osteotomy Preparation:** Implant sites were prepared according to the manufacturer’s protocol.
5. **Implant Placement:** Standardized two-piece dental implants (3.5–4.0.5.0 mm diameter, 10 mm length; ConicalFit, Nuvo Implant, Brazil) were placed subcrestally with an insertion torque $\ge$ 35 Ncm to ensure primary stability. Sites were sutured with interrupted 5-0 silk. All procedures were performed by a single experienced prosthodontist with over 10 years of implant surgery experience and certification in laser therapy, who also served as the principal investigator and first author of this study.

As age and procedure duration are potential confounding factors influencing postoperative pain, the procedure duration was recorded using a timer, starting from the initial incision for flap elevation and ending upon completion of suturing. The duration for all cases ranged between 20 and 25 minutes. Age was documented and statistically analyzed to assess any significant differences between groups. The potential impact of these variables on the study outcomes is further addressed in the Results.

**Fig. 1:**
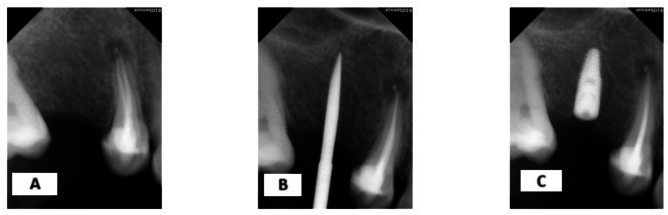
Preoperative, intraoperative, and postoperative periapical radiographs showing implant site status and placement accuracy.

**Fig. 2:**
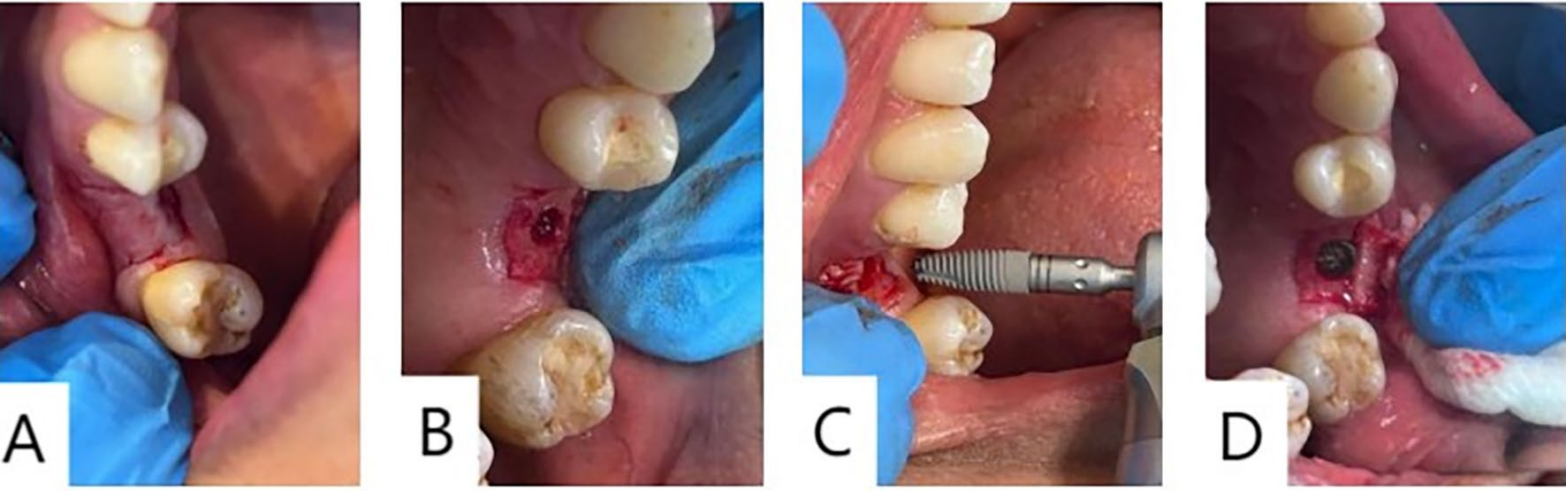
Sequential intraoral clinical images illustrating surgical steps: (**A**) crestal H-shaped flap incision, (**B**) osteotomy preparation, and (**C**-**D**) implant placement.

The standardized surgical steps, from preoperative evaluation to implant placement, are visualized in Figs. 1 and 2.

#### Interventions: laser therapy

**Fig. 3:**
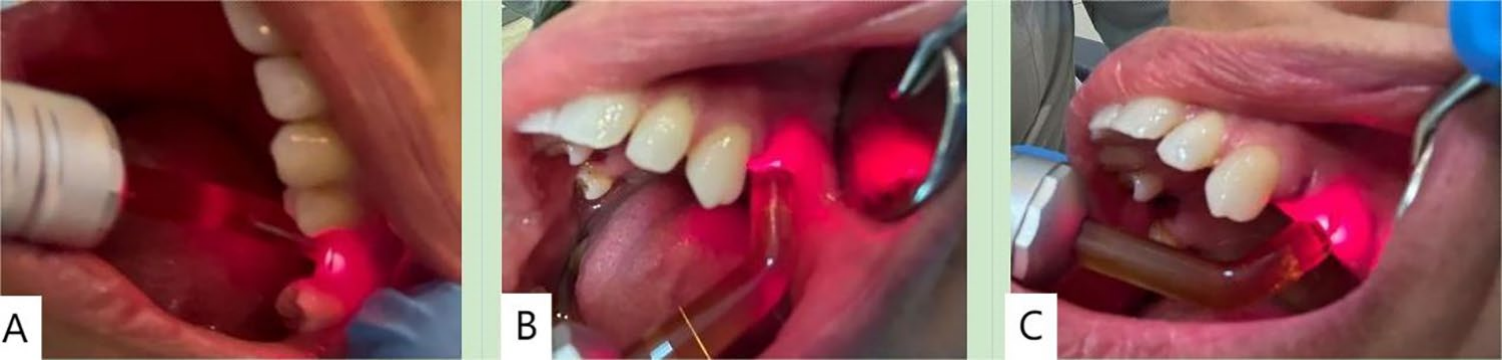
Photobiomodulation protocol application points: crestal, buccal, and palatal aspects of the implant site.

Laser therapy was performed immediately after implant placement and flap suturing at three peri-implant points: buccal, palatal, and crestal, as illustrated in Fig. 3. A probe with a 0.5 cm² diameter was applied, using a gentle contact with the mucosal surface during irradiation. The laser devices used in the study, operating at wavelengths of 650 nm and 810 nm, are described in detail in Table1. Participants in the Sham Laser Group received simulated laser therapy where the probe was positioned identically at each site for 60 seconds per point. To maintain blinding, a mobile phone was used to play the characteristic sound of laser emission to accurately simulate the active treatment environment.

Adverse events were monitored at each follow-up visit. Postoperative pain was managed with acetaminophen, which was recorded as part of the outcome measures. No serious adverse events were anticipated or observed.

**Table 1:** Specifications and Laser Parameters for 650 nm and 810 nm Diode Laser Groups.

	810 nm Laser (Group A)	650 nm Laser (Group B)
Laser Type	Diode Laser	Diode Laser
Active Medium	Gallium Aluminum Arsenide (GaAlAs)	Indium Gallium Phosphide (InGaP)
Maximum Power Output	5000 mW	100 mW
Available Modes of Emission**	Pulsed & Continuous Wave	Continuous Wave
Beam Profile	Gaussian	Gaussian
Manufacturer	Elexxion, Germany	NILES, Egypt*
Wavelength	Infra-Red: 810 nm	Red: 650 nm
Power Output	100 mW (0.1 W)	100 mW (0.1 W)
Exposure Duration/point (s)	60 s	60 s
Energy per point (J)	6 J	6 J
Energy Density *fluence*/point ($\hbox {J/cm}^2$)	12$\hbox {J/cm}^2$	12$\hbox {J/cm}^2$
Emission Mode Used in Study**	Continuous	Continuous
Spot Size ($\hbox {cm}^2$)	0.5	0.5
Irradiated Points	3 (occlusal, buccal, palatal)	3 (occlusal, buccal, palatal)
Total Radiant Energy (J)	18 J	18 J
Total Energy Density ($\hbox {J/cm}^2$)	36$\hbox {J/cm}^2$	36$\hbox {J/cm}^2$
Application Technique	Static Punch mode - contact	Static Punch mode - contact
Treatment Sessions	6 sessions, 3 sessions/week	6 sessions, 3 sessions/week

*Device manufactured and calibrated by Dept. of Laser Applications in Engineering, National Institute of LASER Enhanced Sciences (NILES), Cairo University, Egypt.

**The Available Emission Modes details the manufacturer-specified options of the device, while the Emission Mode Used in Study specifies the setting selected for the treatment protocol in this clinical trial.

### Outcome measures

#### Primary outcome

Pain intensity was measured using an 11-point Numeric Rating Scale (NRS) at 2, 6, 12, 24, 48, and 72 hours postoperatively. In this study, participants were trained to use the NRS for pain assessment to ensure consistency and accuracy in self-reporting. The NRS is a validated, straightforward tool where individuals rate their pain on a scale from 0 to 10, with the following anchors shown in Table 2.

Using standardized recording sheets (Supplementary File 2), participants documented the maximum pain intensity experienced since the previous time point at each specified interval.

**Table 2:** Numerical Rating Scale (NRS) for Pain Assessment.

NRS Score	Pain Level	Description
0	No pain	No sensation of pain
1–3	Mild pain	Nagging, annoying, but manageable; does not interfere with daily activities
4–6	Moderate pain	Distressing, distracting; may interfere with daily activities and concentration
7–9	Severe pain	Intense, dominating focus; limits physical activity and functioning
10	Unbearable pain	The most intense pain imaginable; completely incapacitating

Pain data were extracted and analyzed at two distinct levels:

1. **Temporal Analysis of Pain Scores** Mean pain scores were compared at two temporal scales:
  **Daily Pain Evolution:** Mean scores were compared across the three postoperative days (Day 1: 0–24 h, Day 2: 24–48 h, and Day 3: 48–72 h). For this analysis, the daily pain score was defined as the highest Numerical Rating Scale (NRS) value recorded within its respective 24-hour interval.
  **Early Postoperative Dynamics:** A detailed time-specific assessment was performed using NRS scores recorded at 2, 6, 12, and 24 hours on Day 1 to explore the acute recovery phase.
  The data collection sheet included time-specific NRS entries at 2 h, 6 h, 12 h, 24 h, 48 h, and 72 h postoperatively. Each recorded score reflects the highest level of pain perceived by the patient within the preceding time window (e.g., 0–2 h, 2–6 h, etc.), rather than a single point-in-time measurement. This time-window-based approach ensured standardized and meaningful daily comparisons across patients and treatment groups Supplementary File 2.
2. **Analysis of Pain Severity Over Time** Pain NRS scores were categorized into five ordinal levels to reflect clinical relevance: 0 = No pain, 1 to 3 = Mild pain, 4 to 6 = Moderate pain, 7 to 9 = Severe pain and 10 = Unbearable pain (treatment failure)^20^. The frequency and proportion of each category of severity of pain were calculated for each study group (Sham, PBM 650 nm, and PBM 810 nm) at each recorded time point (2, 6, 12, 24, 48, and 72 hours).

#### Secondary outcomes

1. **Analgesic intake:** Participants received structured training to ensure accurate and transparent reporting of analgesic consumption, with reporting intervals aligned with those of the NRS pain assessments. At each interval, participants documented whether they had taken a rescue dose of 1000 mg dose of acetaminophen within the prior 4 hours (Yes/No). This binary outcome was used to correlate pain scores with analgesic consumption and to assess the frequency of rescue medication use across study groups. They were instructed to use acetaminophen 1000 mg only if their pain score exceeded 3 on the NRS, indicating moderate or higher pain. A log sheet Supplementary File 2 was provided along with verbal instructions. The training aimed to standardize pain score reporting and analgesic use, and minimize the risk of misreporting, underreporting, or unnecessary medication intake^21^. Analgesic intake was evaluated as total and preemptive use. Total intake represents the proportion of participants who consumed any analgesics at each postoperative time point, whereas preemptive intake refers to administration before a pain score of NRS $\ge$ 4, serving as an indicator for assessing the validity of analgesic outcomes.
2. **Oral health-related quality of life (OHRQoL) Assessment: ** OHRQoL was assessed using the **OHIP-14** (Supplementary File 3) at baseline (before surgical intervention), 1 week post-implant placement, and 1 week post-prosthesis placement. The OHIP-14 questionnaire was administered electronically via Google Forms, which were delivered through WhatsApp to the patients. Participants completed the forms independently, and their responses were collected and analyzed to evaluate changes in OHRQoL at the specified time points.

### Statistical analysis

Pain scores were tested for normality using the Shapiro-Wilk test. Raw NRS pain scores (0–10) were treated as continuous data, while categorized pain scores (no pain to unbearable pain) were considered ordinal data. Depending on data distribution, comparisons among groups were performed using either repeated-measures ANOVA (for parametric data) or the Kruskal-Wallis test (for non-parametric data). Effect sizes were calculated and reported as Cohen’s f for overall ANOVA and Cohen’s d for pairwise comparisons. Effect size interpretation followed standard thresholds: small ($d = 0.2, f = 0.1$), medium ($d = 0.5, f = 0.25$), and large ($d \ge 0.8$, $f \ge 0.4$). Analgesic intake (categorical data) was analyzed using the Chi-square test of independence. Where small expected frequencies were observed, Monte Carlo simulation was used for accurate p-value estimation.

Statistical significance was set at $p < 0.05$. All analyses were conducted using IBM SPSS Statistics version 25. Continuous variables were reported as mean ± standard deviation (SD) or median (interquartile range, IQR), and categorical data as frequencies (%). All participants were analyzed in their originally assigned groups (intention-to-treat analysis). No missing data were present. No subgroup or unplanned post hoc analyses were performed.

**Fig. 4:**
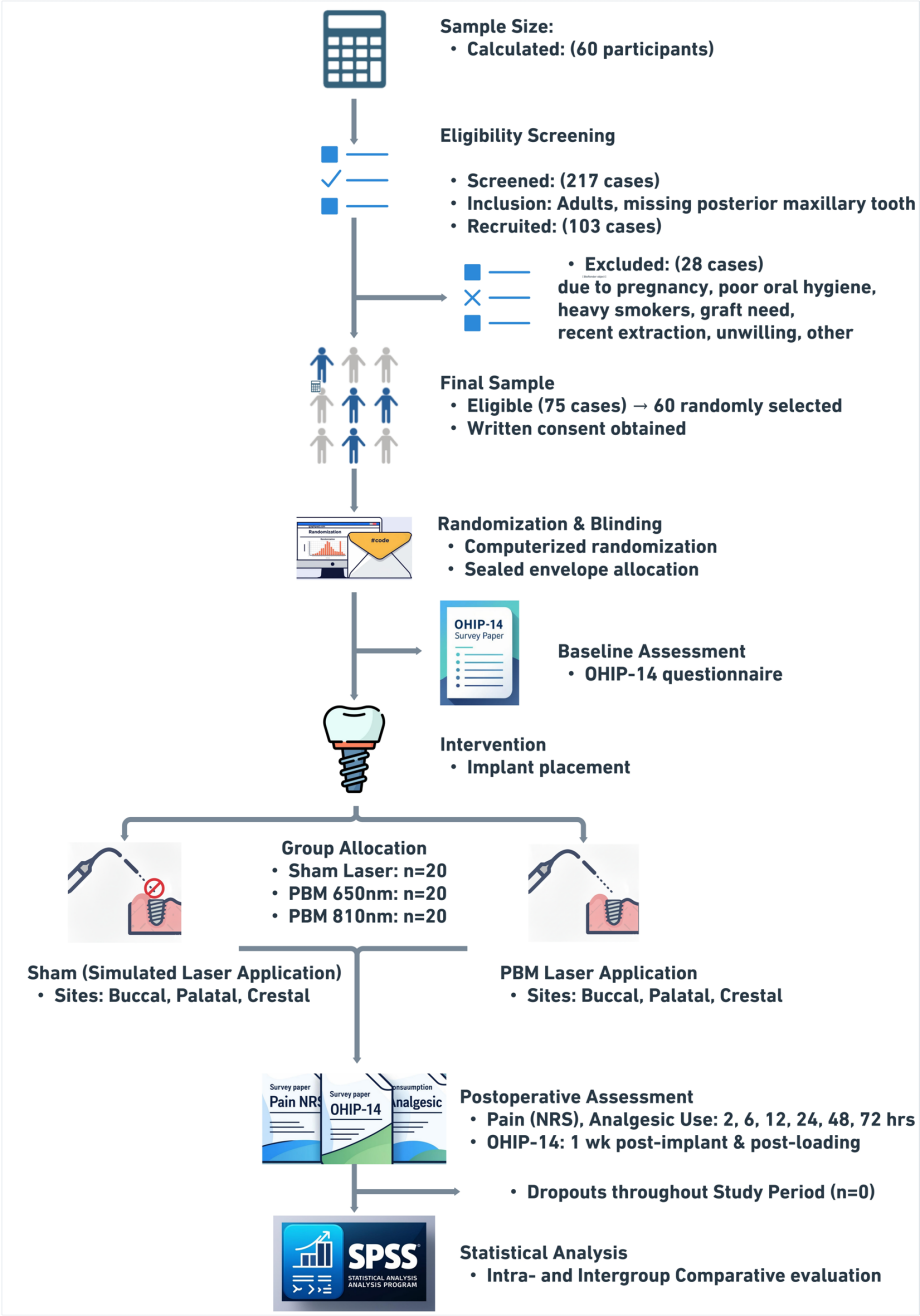
Infographic Step-by-Step Study Trial.

An infographic outlining the step-by-step study protocol, including recruitment, randomization, intervention, and outcome assessments, is provided in Fig. 4. The trial was completed as planned and was not terminated early. A detailed trial protocol and statistical analysis plan have been submitted to the journal as supplementary files and are available upon request.

## Results

The final study sample was composed of 60 patients, of which 50% (n = 30) were men and 50% (n = 30) were women, with ages ranging between 20 and 65 years, and no significant differences were observed between treatment groups in the demographic and clinical variables, ensuring the comparability of the groups at baseline (Table3). No harms or unintended events occurred in any group throughout the study period. All participants were included in the final analysis. Outcome data were available for all participants at each follow-up.

**Table 3:** Demographic Distribution among Study Groups.

Variable	Sham Group (n=20)	PBM 650 nm (n=20)	PBM 810 nm (n=20)	***p-value***
Mean Age (years)	39.5 ± 6.1	38.8 ± 5.7	40.2 ± 5.9	0.73
Male/Female ratio	10/10	10/10	10/10	1.00

Values are presented as mean ± standard deviation. Group comparisons were analyzed using one-way ANOVA. Significance set at p < 0.05.

### Primary outcome

1. **Temporal Analysis of Pain Scores:**
  **Daily Pain Evolution (Days 1–3):** Pain scores (NRS) decreased significantly over time in all groups, with the PBM groups showing faster early relief. On Day 1, pain was significantly higher in the sham group (4.45 ± 2.6) compared to PBM 650 nm (2.4 ± 1.5) and 810 nm (2.8 ± 1.7) (p = 0.004), with a significant overall group effect (p = 0.004) and a large effect size (Cohen’s f = 0.45). Post hoc comparisons revealed a large and statistically significant difference between sham and PBM 650 nm (p = 0.005, Cohen’s d = 0.97) as well as a medium-to-large and significant difference between sham and PBM 810 nm (p = 0.03, d = 0.76). The difference between the two PBM groups was not statistically significant (p = 0.8) and showed a small effect size (d = 0.25). No significant intergroup differences were found on Days 2 and 3 (p > 0.05) as pain resolved in all groups (Table 4; Fig. 5). All groups demonstrated statistically significant reductions in pain over time (p < 0.001) with large effect sizes.

**Table 4:** Daily Pain NRS Scores Treatment Group.

Groups	Sham	PBM 650nm	PBM 810nm	*p*-value	Pairwise comparisons (Day 1)
Days	Mean (SD)	Mean (SD)	Mean (SD)		
**Day 1**	4.45 (2.58)	2.4 (1.50)	2.8 (1.70)	**0.004**	$p_1$**= 0.005**,$d_1 = 0.97$(Sham vs PBM 650 nm)
					$p_2$**= 0.03**,$d_2 = 0.76$(Sham vs PBM 810 nm)
					$p_3 = 0.80$,$d_3 = 0.25$(PBM 650 nm vs PBM 810 nm)
**Day 2**	0.9 (2.02)	0.1 (0.45)	0.45 (1.36)	0.22	–
**Day 3**	0.7 (1.84)	0.1 (0.45)	0.25 (0.72)	0.25	–
***p***-value	$<{\textbf { 0.001}}$	$< {\textbf {0.001}}$	$<{\textbf { 0.001}}$	–	–

Values are presented as mean ± standard deviation. One-way ANOVA was used for between-group comparisons. Post hoc pairwise tests with Cohen’s *d* were used for Day 1. Statistical significance was set at$p < 0.05$.

$p_1$/$d_1$: Sham vs PBM 650 nm;$p_2$/$d_2$: Sham vs PBM 810 nm;$p_3$/$d_3$: PBM 650 nm vs PBM 810 nm.
    **Fig. 5:**
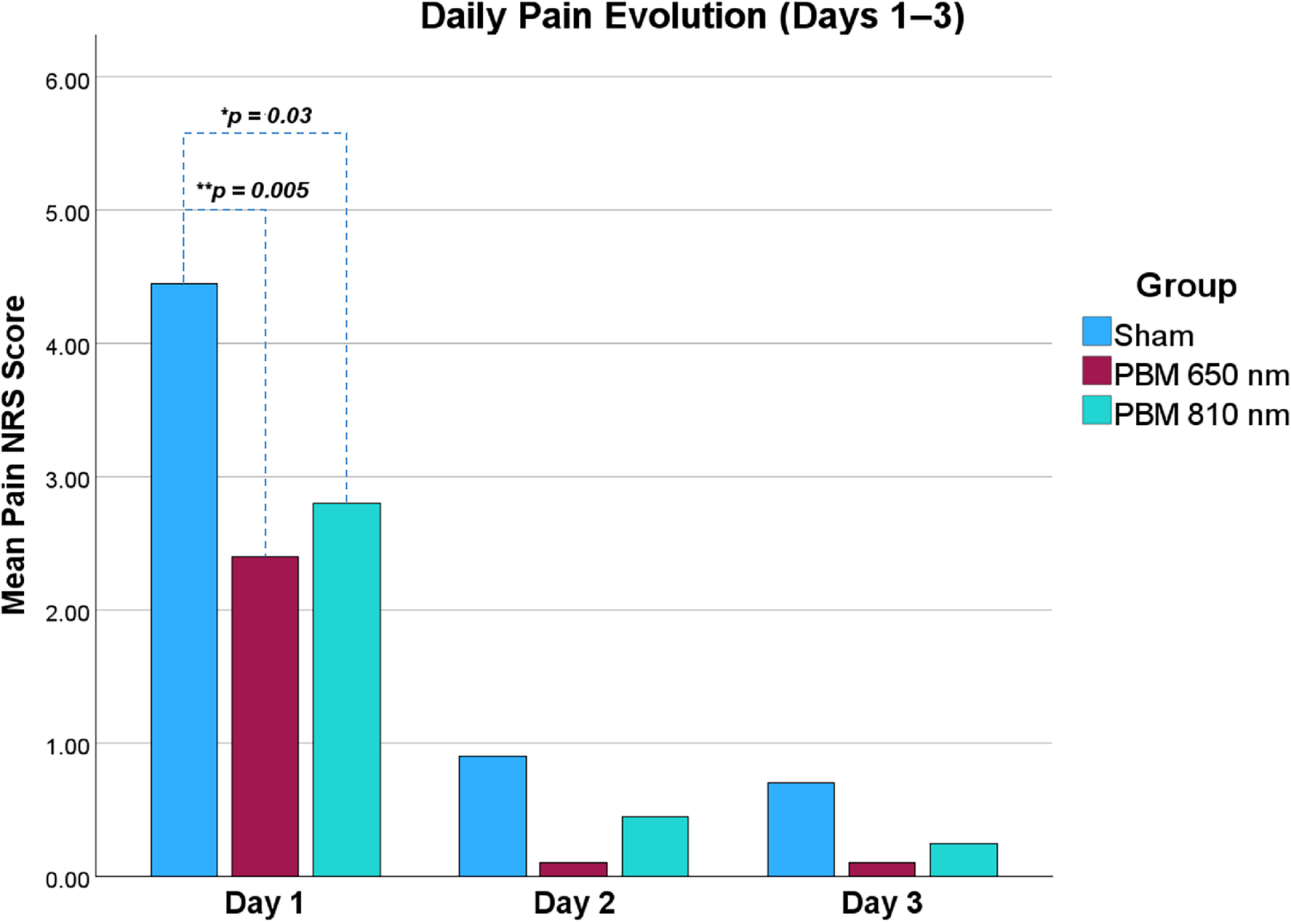
Bar chart depicting the daily evolution of mean pain scores (NRS) across the Sham, PBM 650 nm, and PBM 810 nm groups over the 3-day study period. The Sham group exhibited significantly higher pain scores on Day 1 compared to both PBM groups (p = 0.004), while all groups showed a progressive decline by Day 3.
  **Early Postoperative Pain (2–24 Hours): ** At 2 hours, pain scores were significantly lower in both the 650 nm (2.35 ± 1.53) and 810 nm (2.65 ± 1.53) groups compared to the sham group (4.40 ± 2.52), with a statistically significant group effect (p = 0.003; one-way ANOVA). The effect size was large ($\eta ^2 = 0.19$, Cohen’s $f = 0.47$), indicating that approximately 19% of the variance in early postoperative pain could be attributed to group differences. From 6 to 24 hours, all groups showed further reduction in pain with non-significant differences (Table 5; Fig. 6).

**Table 5:** Pain NRS Scores at 2, 6, 12, and 24 Hours Post-Treatment on Day 1 with Intergroup Statistical Comparisons.

Timepoint	650 nm	810 nm	Sham	***p-value***
2h	2.35 ± 1.53	2.65 ± 1.53	4.4 ± 2.52	**0.003**
6h	1.35 ± 1.72	1.85 ± 1.98	3.0 ± 2.52	0.08
12h	0.8 ± 1.01	1.1 ± 1.29	1.95 ± 2.31	0.08
24h	0.25 ± 0.72	0.8 ± 1.44	1.4 ± 2.52	0.12

**Fig. 7:**
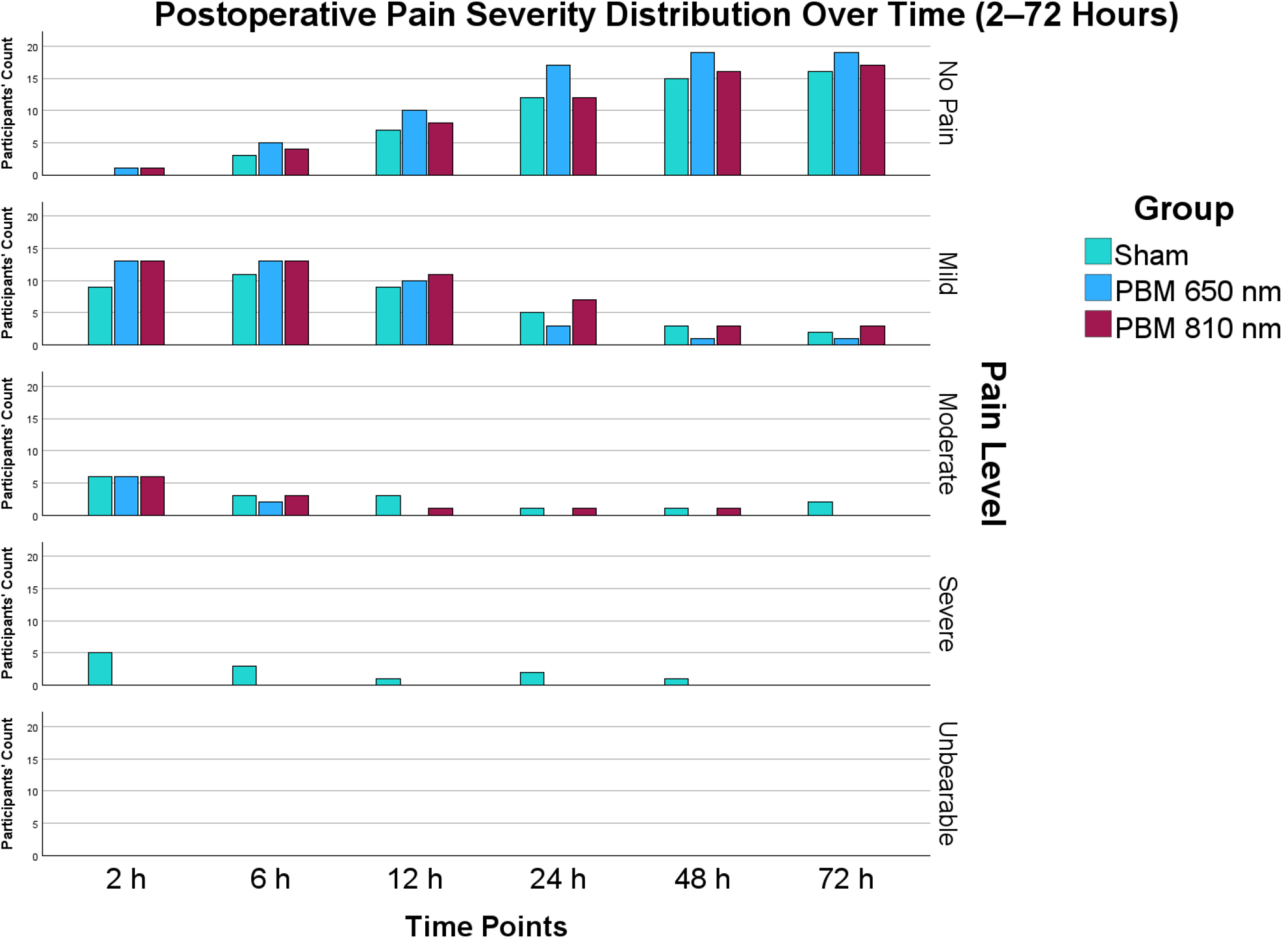
Comparative Pain Severity Outcomes Across Treatment Groups Over 72 Hours. Clustered bar chart illustrating the distribution of participants by categorized pain level (NRS 0–10) at six postoperative time points. The data confirm that Photobiomodulation (PBM) prevents extreme pain events: 0 participants in either PBM group reported Severe or Unbearable pain across the 72-hour period. Furthermore, the PBM 650 nm group demonstrates superior early and sustained pain control, leading the No Pain category and showing a rapid clearance from the Moderate pain level compared to the Sham control.

Values are presented as mean ± SD. One-way ANOVA was used for between-group comparisons. *p* < 0.05 was considered significant.
  **Fig. 6:**
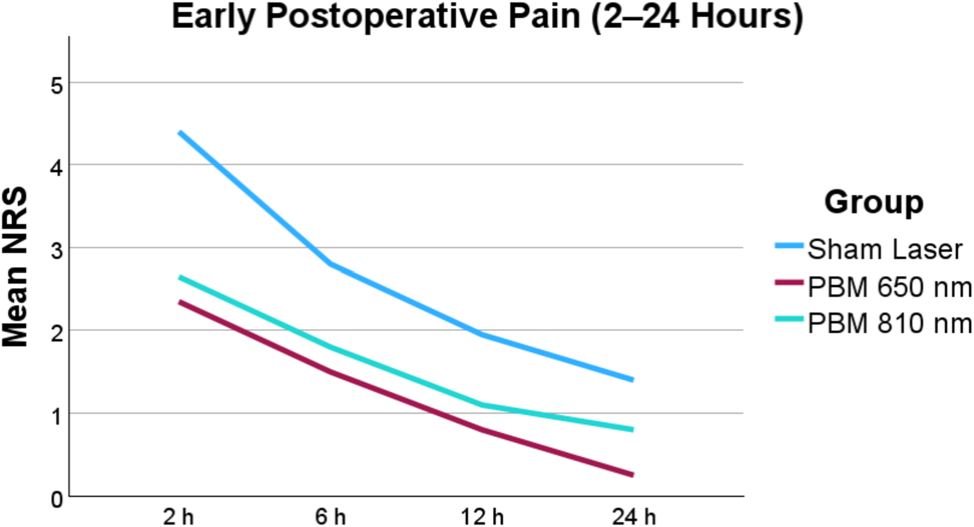
Temporal pain trends within the first 24 hours postoperatively, illustrating mean NRS scores at 2, 6, 12, and 24 hours for each group. Significant intergroup differences were observed at 2 hours (p = 0.003), with the PBM 650 nm and PBM 810 nm groups reporting lower pain compared to the Sham group. Pain levels declined across all groups over time.
2. **Pain Severity Over Time:** At 2 hours, severe pain (NRS 7–9) was reported in 25% of sham patients but absent in both PBM groups (p $\approx$ 0.02–0.04). The sham group continued to show a higher incidence of moderate/severe pain through 24 hours, though differences became less pronounced over time (Table 6; Fig. 7). By 24 hours, 85% of PBM 650 nm patients reported no pain, compared to 60% in the sham and 810 nm groups.

**Table 6:** Distribution of Pain Severity Scores Among Study Groups During the First 24 Hours Postoperatively.

Pain intensity	[NRS]	No Pain [0]	Mild Pain [1–3]	Moderate Pain [4–6]	Severe Pain [7–9]	Unbearable Pain [10]	Total cases
Time point	Laser Group	N (%)	N (%)	N (%)	N (%)	N (%)	N
**2hr**	**Sham**	0 (0%)	9 (45%)	6 (30%)	**5 (25%)**	0 (0%)	20
	**650**	1 (5%)	13 (65%)	6 (30%)	**0 (0%)**	0 (0%)	20
	**810**	1 (5%)	13 (65%)	6 (30%)	**0 (0%)**	0 (0%)	20
**6hr**	**Sham**	3 (15%)	11 (55%)	3 (15%)	**3 (15%)**	0 (0%)	20
	**650**	5 (25%)	13 (65%)	2 (10%)	**0 (0%)**	0 (0%)	20
	**810**	4 (20%)	13 (65%)	3 (15%)	**0 (0%)**	0 (0%)	20
**12hr**	**Sham**	7 (35%)	9 (45%)	3 (15%)	**1 (5%)**	0 (0%)	20
	**650**	10 (50%)	10 (50%)	0 (0%)	**0 (0%)**	0 (0%)	20
	**810**	8 (40%)	11 (55%)	1 (5%)	**0 (0%)**	0 (0%)	20
**24hr**	**Sham**	**12 (60%)**	5 (25%)	1 (5%)	**2 (10%)**	0 (0%)	20
	**650**	**17 (85%)**	3 (15%)	0 (0%)	**0 (0%)**	0 (0%)	20
	**810**	**12 (60%)**	7 (35%)	1 (5%)	**0 (0%)**	0 (0%)	20

Data presentation of pain severity categories distribution among groups. Values are presented as the number of patients (*N*) and percentage (%).

#### Secondary outcomes

1. **Analgesic Intake** Analgesic use was highest at 2 hours postoperatively (80 % in the sham group vs. 50 % and 65 % in the PBM 650 nm and 810 nm groups, respectively) and declined progressively across all groups thereafter. The sham group consistently demonstrated the highest total analgesic consumption at all time points and the lowest proportion of preemptive (non-compliant) use. Significant differences were observed between the sham and PBM 650 nm groups at 2 hours (P = 0.04) and 6 hours (P = 0.03), while the PBM 810 nm group showed a near-significant reduction compared with sham at 6 hours (P = 0.06). No other intergroup comparisons reached statistical significance. A detailed summary of total and preemptive analgesic intake across postoperative intervals is presented in table 7 and Fig. 8.

**Table 7:** Total and Preemptive Analgesic Intake at Postoperative Time Intervals in Sham and PBM Groups.

Time Point	Sham		PBM 650 nm		PBM 810 nm		$\boldsymbol{P}_{1}$	$\boldsymbol{P}_{2}$	$\boldsymbol{P}_{3}$
	Total n (%)	Preemptive n(%)	Total n (%)	Preemptive n(%)	Total n (%)	Preemptive n(%)			
2 hours	16 (80)	5 (31)	10 (50)	4 (40)	13 (65)	7 (54)	0.04*	0.34	0.29
6 hours	13 (65)	8 (62)	6 (30)	4 (67)	7 (35)	5 (71)	0.03*	0.06	0.74
12 hours	7 (35)	4 (57)	4 (20)	4 (100)	4 (20)	4 (100)	0.29	1.00	0.29
24 hours	5 (25)	3 (60)	1 (5)	1 (100)	2 (10)	2 (100)	0.08	0.55	0.21
48 hours	3 (15)	2 (67)	1 (5)	1 (100)	1 (5)	1 (100)	0.30	1.00	0.30
72 hours	2 (10)	1 (50)	1 (5)	1 (100)	0 (0)	–	0.55	0.31	0.15

**Fig. 9:**
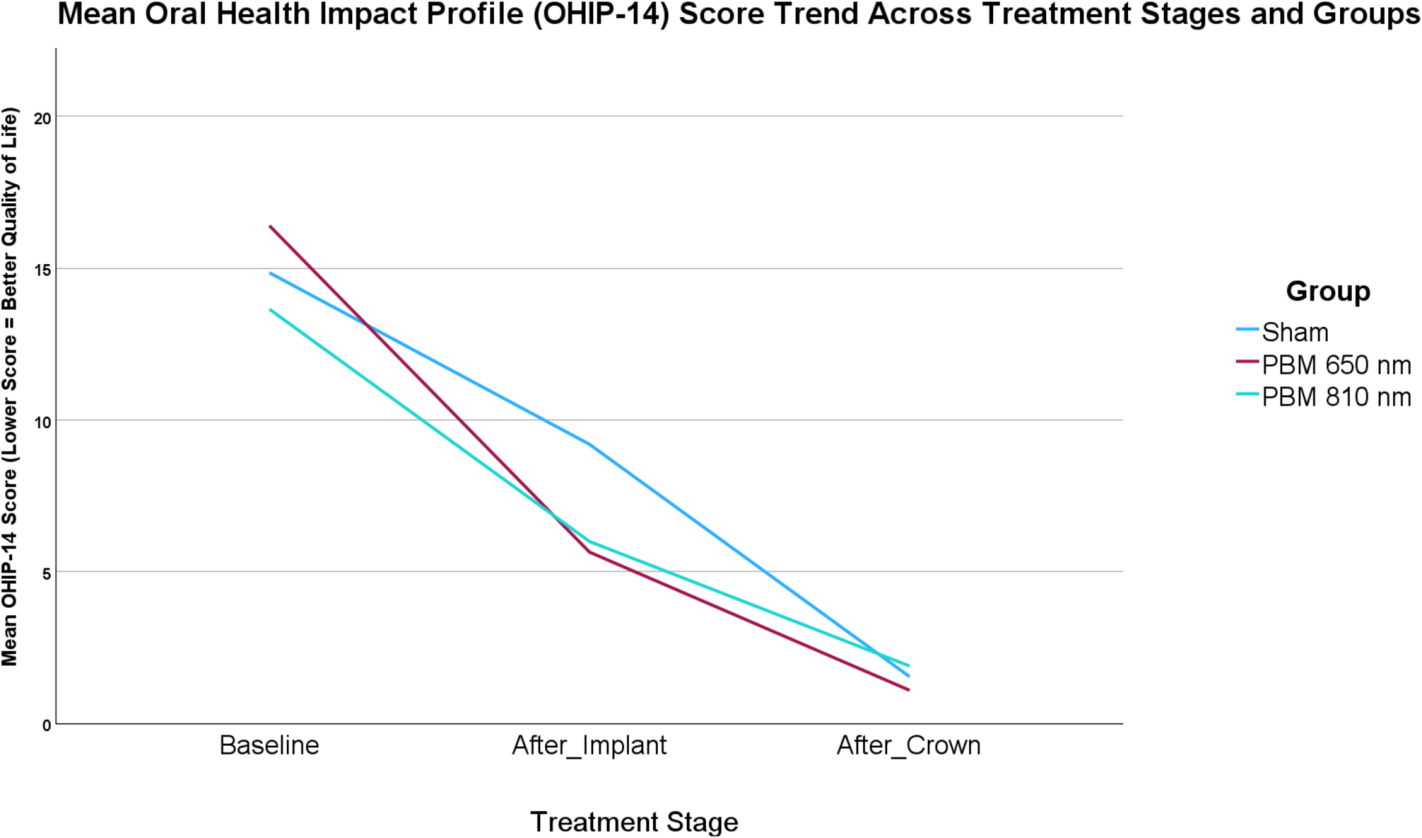
Line chart illustrating the trend of mean OHIP-14 scores across the three time points. All groups demonstrated significant intra-group improvements, although inter-group differences were not statistically significant.

**Fig. 10:**
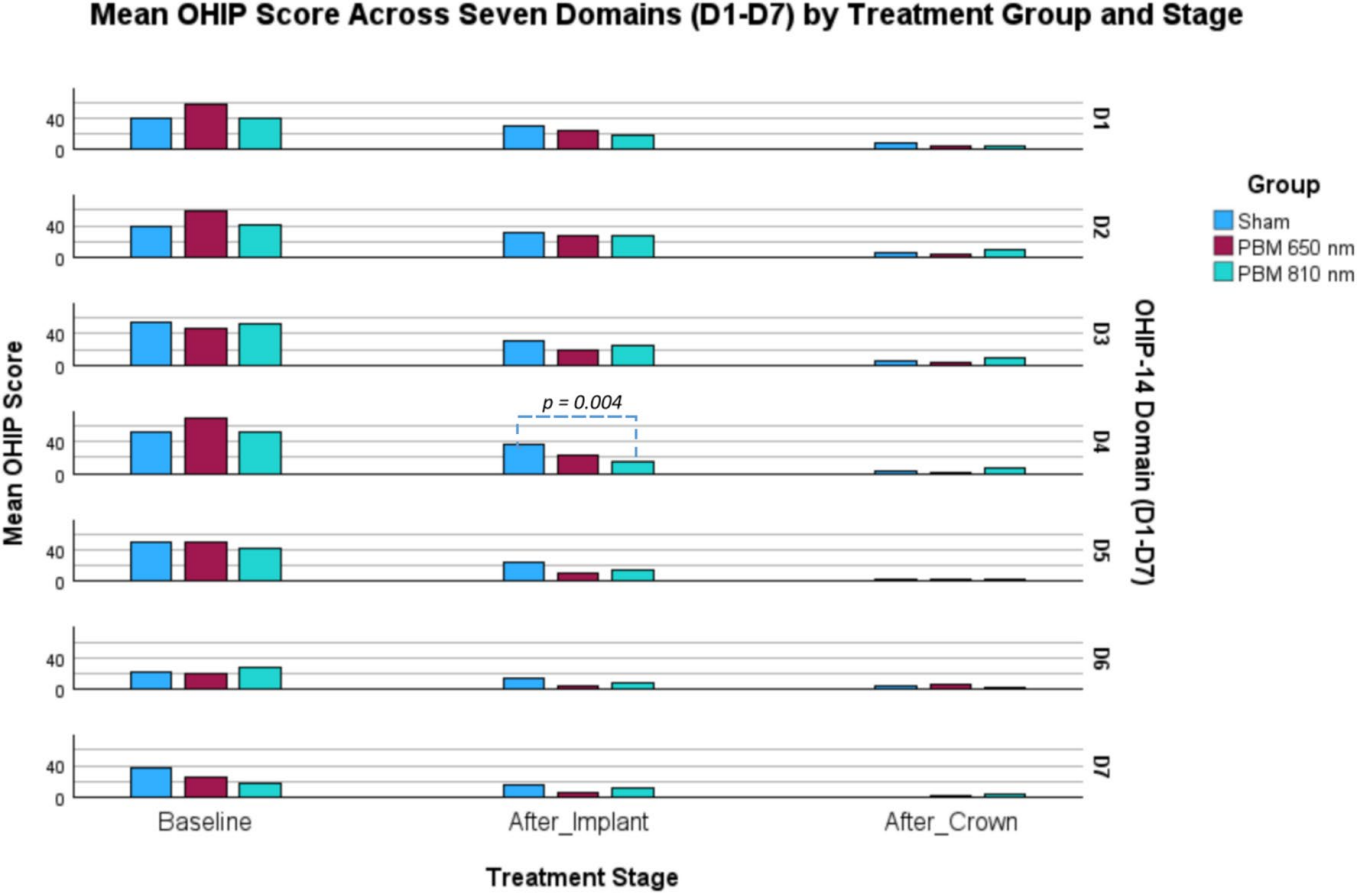
Multi-panel clustered bar chart illustrating the mean score for each of the seven Oral Health Impact Profile (OHIP-14) domains across three time points: Baseline, 1 week Post-Implant, and 1 week Post-Crown delivery. The domains ($\text {D1}$ through $\text {D7}$) represent: $\text {D1}$ (Functional Limitation), $\text {D2}$ (Physical Pain), $\text {D3}$ (Psychological Discomfort), $\text {D4}$ (Physical Disability), $\text {D5}$ (Psychological Disability), $\text {D6}$ (Social Disability), and $\text {D7}$ (Handicap). The PBM 810 nm group demonstrated a statistically significant reduction in the Physical Disability dimension (p = 0.004), indicating improved short-term functional outcomes.

Group comparisons were analyzed using the Chi-square test of independence. When expected cell counts were low, Monte Carlo simulation was applied to estimate *p*-values. *p* < 0.05 was considered statistically significant. $\boldsymbol{P}_{1}$ = Sham vs PBM 650 nm; $\boldsymbol{P}_{2}$ = Sham vs PBM 810 nm; $\boldsymbol{P}_{3}$ = PBM 650 nm vs PBM 810 nm. *Significant at *p* < 0.05. **Total** = total participants taking analgesics. The percentage reported in the **Total** column represents total analgesic users as a proportion of all participants within each group. **Preemptive n** = participants taking analgesics at NRS < 4. **Preemptive %** = percentage of participants taking analgesics at NRS < 4 relative to total analgesic users.
  **Fig. 8:**
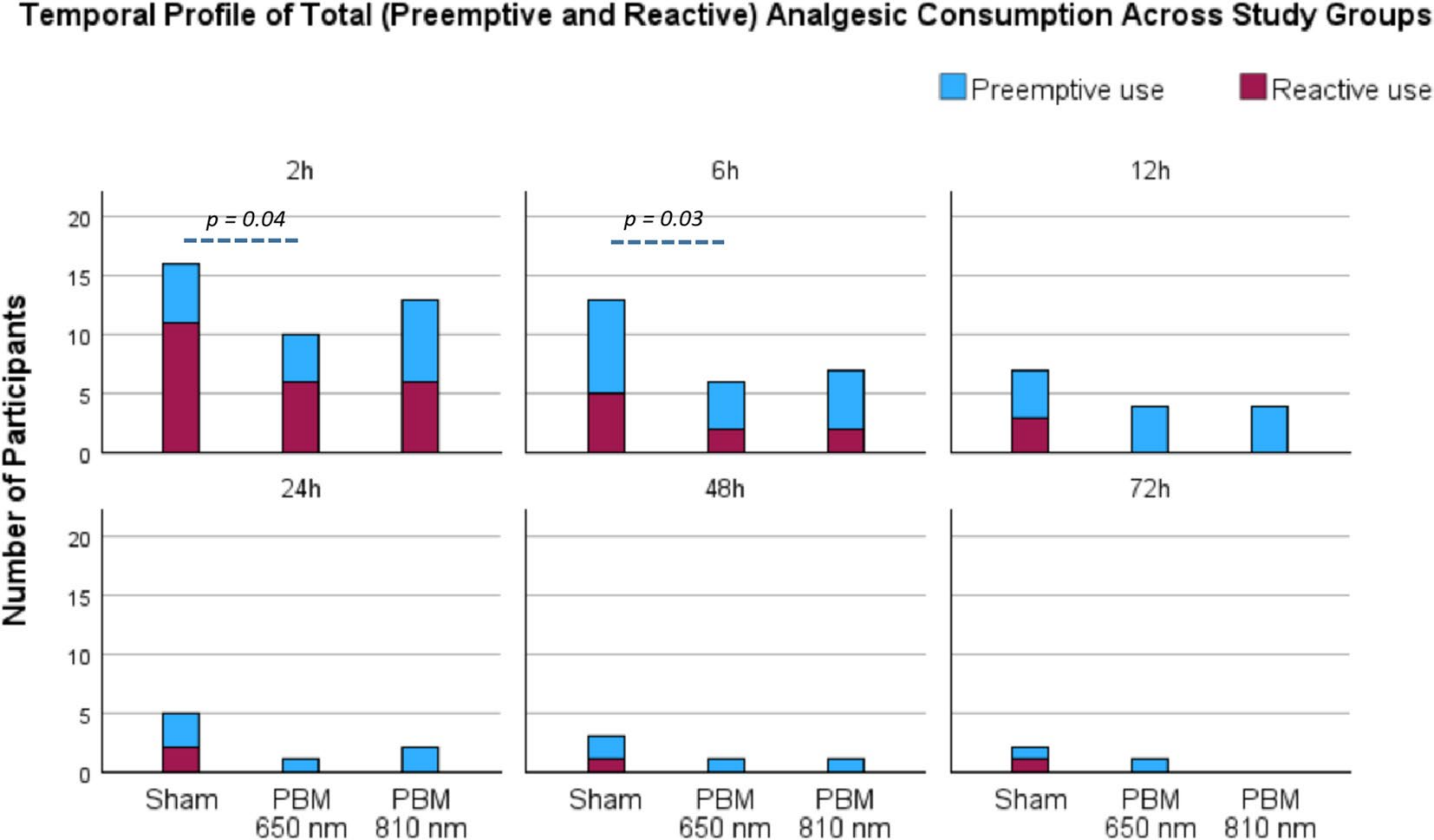
Total analgesic intake and its composition at postoperative time points. Each bar represents the total number of participants in each group who reported analgesic consumption, subdivided into Reactive Use (intake in response to pain, NRS $\ge$ 4) and Preemptive Use (intake at NRS < 4). The trend reflects the temporal decline in pain in all groups, with faster reduction in analgesic use in PBM groups. P-values (p < 0.05, Chi-square with Monte Carlo) denote significant differences in total intake between PBM 650 nm and sham groups.
2. **Oral Health-Related Quality of Life (OHRQoL) ** All groups showed significant within-group improvements in OHIP-14 scores from baseline to post-crown stage (p < 0.01), but no significant differences between groups at any time point (Table 8; Fig. 9). However, when comparing the scores across the seven dimensions of the OHIP-14 one week after implant placement, the PBM 810 nm Group demonstrated a statistically significant reduction in the Physical Disability dimension scores (p = 0.004). This comparison is illustrated in Fig. 10.

**Table 8:** Mean OHIP-14 Scores at Baseline, After Implant Placement, and After Crown Placement in Sham, PBM 650 nm, and PBM 810 nm Groups.

OHIP-14 Score	Sham Group (n=20)	PBM 650nm Group (n=20)	PBM 810 nm Group (n=20)	***p-value***
**Baseline**	7.45 (2.89)	8.21 (2.14)	6.81 (2.62)	*0.23*
**After implant placement**	4.57 (4.25)	2.79 (3.25)	3.00 (3.38)	*0.25*
**After crown placement**	0.76 (1.01)	0.58 (1.27)	0.93 (1.59)	*0.70*
***P-*** ***value***	***P1=*** ***0.013*** ***P2= 1.45e-8***	***P1=*** ***2.14e-7***	***P1=*** ***0.002***	–
**OHIP-14** **C** **hange**($\boldsymbol{\Delta }{{\textbf {OHIP-14}}}$)				
**(Baseline - after implant placement)**	2.89 (4.69)	5.43 (3.08)	3.82 (4.66)	*0.19*
(B**aseline - after crown Placement**)	6.70 (3.19)	7.64 (2.27)	5.89 (3.34)	*0.17*

Values are presented as mean ± standard deviation. Between-group comparisons were performed using one-way ANOVA. Within-group changes over time were analyzed using repeated measures ANOVA. ***P***1: Baseline vs. Post-Implant; ***P***2: Baseline vs. Post-Crown. Statistical significance set at *p* < 0.05.

## Discussion

This randomized controlled trial evaluated the short-term efficacy of PBM using 650 nm and 810 nm diode lasers in reducing postoperative pain and analgesic consumption and enhancing OHRQoL after posterior maxillary dental implant placement. Both wavelengths showed significant early benefits, with 650 nm achieving the most consistent pain control and reduction in medication demand.

Effective postoperative pain management is essential in implant dentistry, particularly in the acute postoperative phase^22^. Our results demonstrated that PBM at both 650 nm and 810 nm wavelengths significantly reduced pain intensity on the first postoperative day compared to sham treatment, with large effect sizes indicating clinically meaningful benefits (Cohen’s d = 0.97 and 0.76; p = 0.005 and 0.03, respectively). There was no significant difference between the two laser groups (p = 0.80, d = 0.25), suggesting comparable analgesic efficacy.

By Days 2 and 3, pain levels decreased in all groups, with no statistically significant differences observed (p > 0.20), indicating that the primary benefit of PBM occurs during the acute phase.

At the early 2-hour postoperative assessment, a significant overall group effect was observed (p = 0.003), with a large effect size ($\eta ^{2}$ = 0.19, Cohen’s f = 0.47), highlighting that approximately 19% of the variance in pain was attributable to treatment. The negligible effect size between the 650 nm and 810 nm groups (f = 0.06) further supports their similar clinical effectiveness. These findings align with previous studies highlighting PBM’s anti-inflammatory and analgesic mechanisms^1,5,6^.

Categorizing pain severity added clinical relevance beyond numerical pain scores. Notably, no participants in the PBM groups reported severe pain, in contrast to 25% in the sham group at the 2-hour mark. By 24 hours, 85% of the 650 nm group were pain-free, compared to 60% in both the 810 nm and sham groups, supporting the clinical potential of superficial-wavelength PBM for acute symptom relief^2,3,23^.

Analgesic consumption was significantly lower in the 650 nm group at early time points, while the 810 nm group showed a reduction that did not reach statistical significance. The interpretation of consumption data must consider preemptive, non-protocol analgesic use at $\text {NRS}\le 3$, which breaks the link between pain and medication use, resulting in misleading outcomes. In this study, such non-compliant intake occurred across all groups.

While such non-compliance can confound consumption data, a detailed analysis of its distribution reinforces the robustness of our finding. The proportion of analgesic intake that was non-compliant was consistently lowest in the Sham group compared to the PBM groups. This pattern strongly suggests that the high consumption in the Sham group was predominantly driven by genuine pain, not anxiety, which supports the validity of the observed significant reduction in analgesic demand in the 650 nm group at 2 and 6 hours.

Furthermore, the authenticity of the PBM effect is underscored by the fact that 50% of the PBM 650 nm cohort required no analgesics at any point, yet still exhibited the greatest reduction in pain scores. These combined findings support the potential of PBM–especially at 650 nm–as a non-pharmacological adjunct for postoperative pain management, particularly in patients advised to avoid systemic analgesics. Similar trends have been noted by Caccianiga et al.^24^ and Azizi et al.^25^, though contrasting findings, such as those from Alqutub^10^, emphasize the importance of patient selection and protocol optimization.

Our results are consistent with literature documenting PBM’s efficacy after dental procedures^5,6,16,26–32^, though our design included more frequent pain assessments within the first 72 hours. Interestingly, the most substantial pain relief reported in our study was limited to the 2-hour mark, which contrasts with studies reporting extended analgesic effects^16,32^, possibly due to limited surgical trauma and a minimally invasive technique, which may have reduced the need for sustained PBM effects beyond the immediate postoperative period.

The observed effects are supported by mechanistic insights into PBM’s biological action. Both 650 nm and 810 nm wavelengths stimulate mitochondrial cytochrome c oxidase, enhancing ATP production, cellular respiration, and tissue repair^3,33^. PBM also modulates inflammation by suppressing pro-inflammatory mediators (TNF-$\alpha$, IL-1$\beta$, PGE2) and increasing anti-inflammatory cytokines (IL-10)^34,35^, thereby reducing peripheral nociceptor sensitization. Immediately following surgery, mediators such as bradykinin, Substance P, and prostaglandins amplify acute pain. PBM applied during this window likely dampens this inflammatory burst, consistent with our findings of early pain relief.

A key challenge in PBM implementation is the lack of standardized dosimetry. PBM protocols for managing postoperative dental pain commonly employ diode lasers in the 650–810 nm range, with output powers typically between 30–100 mW, energy densities from 3–100 J/$\hbox {cm}^2$, irradiation times spanning 17–900 seconds, and spot sizes varying by device and application site^36–39^. For example, a single 808 nm, 100 mW diode laser session delivering 3 J per point over 30 seconds has demonstrated significant reductions in pain, edema, and trismus after third molar extraction^37^. Similarly, 810 nm lasers at 100 mW for 60 seconds per point (6J/point) have been used in multi-point intraoral protocols, showing effective pain and edema control post-surgery^38^. Session frequency varies: both single-session and multi-session protocols are reported, with some studies applying PBM immediately postoperatively and others repeating sessions over several days^36–38,40^. While single-session PBM can yield significant short-term pain relief, multi-session approaches may enhance cumulative effects but risk diminishing returns if overdosing occurs^40^.

The biological rationale for PBM dosing is grounded in the wavelength-dependent tissue penetration: longer wavelengths (e.g., 810–830 nm) penetrate deeper, targeting submucosal and periosteal tissues, while shorter wavelengths (e.g., 650–660 nm) are more superficial, influencing mucosal healing^36,38,39^. The biphasic dose response (Arndt-Schulz curve) is central to PBM, where low to moderate doses stimulate cellular activity and analgesia, but excessive dosing may inhibit healing or provide no additional benefit^36,41,42^. This underscores the importance of precise dosimetry, as both under- and overdosing can result in suboptimal or even inhibitory outcomes^36,41^.

In the present study, the more immediate effects observed with the 650 nm wavelength may be attributed to its stronger absorption in superficial tissues, facilitating faster epithelial closure and early nociceptor modulation. Conversely, the deeper penetration of 810 nm light may produce more gradual, sustained effects, particularly in promoting tissue regeneration and angiogenesis^23^. This dual, wavelength-dependent mechanism of action is consistent with findings reported by Birang et al.^5^, and Camolesi et al.^6^

Despite promising results, there remains a lack of consensus on optimal PBM parameters for postoperative dental pain and inflammation, with significant heterogeneity in wavelength, power, energy density, and session frequency across studies^36,40–42^. This variability, along with inconsistent dosimetric reporting, complicates protocol standardization and evidence synthesis^36,41,42^. Strengths of current evidence include the demonstration of PBM’s safety and its potential to reduce pain and analgesic use, but limitations involve small sample sizes, short follow-up, and methodological heterogeneity^36–38,40–42^.

OHRQoL outcomes, assessed via OHIP-14, improved over time in all groups, suggesting a natural recovery effect following implant placement. However, inter-group differences were not statistically significant, limiting conclusions about PBM’s independent contribution to quality of life improvements. These findings should be interpreted with caution, especially given the positive tone of early results. They reflect more on the surgical success and psychological reassurance of treatment than on the distinct effect of PBM.

Nonetheless, dimension-specific improvements–such as the 810 nm group’s significant gain in the ”Physical Disability” domain (p = 0.004)–may hint at wavelength-specific benefits, though these require confirmation in larger samples. The 650 nm group showed faster overall OHIP-14 score decline, potentially reflecting superficial comfort rather than deep-tissue recovery. Similar studies have reported mixed results: while Martins et al.^43^ observed PBM-related OHRQoL gains in radiotherapy-induced mucositis, Moraes et al.^44^ found no such differences in less invasive contexts.

This study’s strengths include a sham-controlled design with sex-stratified randomization, operator blinding, and standardized laser protocols. Pain was assessed using the validated NRS, and OHRQoL was measured with the sensitive weighted OHIP-14^45^. Categorizing pain by severity enhanced clinical relevance. Furthermore, integrating OHRQoL with NRS scores and analgesic use offers a comprehensive, patient-centered assessment of postoperative recovery by capturing both pain intensity and its broader impact on daily life^46^.

Despite its strengths, several limitations should be noted. Pain and OHRQoL were self-reported, introducing subjectivity. The sample size was powered for primary outcomes but insufficient for subgroup analyses or rare events. Surgeon blinding was unfeasible, a limitation mitigated by a fully standardized, time-controlled protocol. Furthermore, behavioral factors were not fully controlled; compliance with analgesic-use instructions varied–some participants took medications preemptively, an action likely confounded by patient anxiety and pain anticipation. Anxiety, a known pain modulator, was not assessed in this study. Finally, while the OHIP-14 is a validated instrument, its application in Egyptian populations lacks specific cultural validation.

Future PBM research should prioritize the harmonization of treatment protocols and the consistent adoption of standardized dosimetric reporting guidelines–ideally aligned with frameworks such as those proposed by the World Association for Laser Therapy (WALT). Incorporating patient-specific factors (e.g., tissue thickness, surgical complexity) and objective clinical markers (e.g., edema, perfusion) will enhance the clinical applicability and personalization of PBM interventions. Further studies are needed to explore multi-wavelength PBM strategies, dose optimization, and session frequency, ideally integrating both subjective outcomes (e.g., pain scores) and objective biomarkers (e.g., cytokine levels, hemodynamic changes). Stratifying participants based on baseline pain intensity and psychological profiles (e.g., anxiety levels, treatment expectations) may also improve the precision and interpretability of outcomes. Notably, the synergistic effects of dual-wavelength PBM protocols (e.g., 650 nm + 810 nm) warrant investigation for potentially superior therapeutic efficacy. Surgeon blinding can be achieved by employing externally controlled devices that deliver active or sham outputs via identical probes. Finally, the incorporation of clinically meaningful thresholds–such as the Minimal Clinically Important Difference (MCID) and Patient Acceptable Symptom State (PASS)–will enhance both the interpretability and translational relevance of future findings^47^.

## Conclusion

In conclusion, this study found that PBM using 650 nm and 810 nm diode lasers effectively reduced acute postoperative pain and analgesic use after single-implant placement. The analgesic benefit was transient, limited primarily to the first few hours post-surgery, with the 650 nm wavelength demonstrating superior short-term analgesic benefit. No adverse effects were observed, supporting PBM’s safety as a potential adjunct for early recovery. However, the absence of significant differences in OHRQoL suggests that its broader patient-centered benefits remain uncertain and warrant further investigation in more invasive surgical contexts.

## Supplementary Information

Supplementary Information 1.

Supplementary Information 2.

Supplementary Information 3.

## Data Availability

The datasets generated and analyzed during the current study are publicly available in the Zenodo repository (https://zenodo.org/records/15956427). The deposited files include the de-identified participant data. The experimental protocol is available at 10.17504/protocols.io.4r3l2zm6jl1y/v1. A preprint of this manuscript is available at 10.21203/rs.3.rs-6858207/v1.
