# Supplementary material for: Analgesic outcomes of 650 nm versus 810 nm diode laser photobiomodulation after dental implant placement in a randomized controlled trial
Source: Sci Rep. 2026 Apr 21;16:19671. doi: 10.1038/s41598-025-32248-8 (PMC13315321; doi:10.1038/s41598-025-32248-8)
Supplement: Supplementary file 1 — Supplementary Information 1. [file 41598_2025_32248_MOESM1_ESM.pdf]

Informed Consent Form for Participation in a Randomized Controlled Trial

Study Title

Comparative Effects of 650 nm and 810 nm Diode Laser Photobiomodulation on Postoperative Pain, Analgesic Use, and OHRQoL After Dental Implant Surgery: A Randomized Controlled Trial

Principal Investigator

Dr. Mohammad Yehya, BDS, MDS, PhD candidate

Researcher at the National Institute of Laser Enhanced Sciences (NILES), Cairo University, Egypt

Facility

This study is being conducted at the clinical facilities of the National Institute of Laser Enhanced Sciences (NILES), Cairo University, Egypt.

Introduction

You are being invited to participate in a clinical research study conducted at the National Institute of Laser Enhanced Sciences (NILES), Cairo University. Before you decide whether to participate, it is important to understand the purpose of the research and what it will involve. This form provides comprehensive information about the study, including its purpose, procedures, risks, and benefits. Please read this form carefully and feel free to ask any questions before signing.

Purpose of the Study

This randomized controlled trial (RCT) aims to evaluate the effectiveness of low-level laser therapy (LLLT) using 650 nm and 810 nm diode lasers on reducing postoperative pain and improving healing outcomes following dental implant placement. The study seeks to determine whether photobiomodulation can enhance recovery when used alongside standard post-implant care.

Eligibility Criteria

You have been invited to participate in this study because you meet the following inclusion criteria:

- Adults aged 20 to 65 years

- Indicated for a single dental implant in the posterior maxilla (premolar or molar region)

- Classified as ASA Physical Status I or II (i.e., healthy or with mild systemic disease)

- In good oral health

- Willing and able to comply with all study procedures and scheduled follow-up visits

- Able to provide written informed consent

You will not be eligible to participate in this study if any of the following exclusion criteria apply:

- Use of analgesics, NSAIDs, or corticosteroids within 48 hours prior to surgery

- History of alcohol abuse or regular consumption exceeding 14 drinks/week for men or 7 drinks/week for women, or alcohol withdrawal within the past 6 months

- History of chronic pain or current long-term pain medication use

- Requirement for grafting, sinus lift, or any complex implant procedure at the study site

- Pregnancy or breastfeeding

- Presence of any uncontrolled systemic disease (e.g., uncontrolled diabetes, immunodeficiency)

- History of epilepsy, photosensitivity, or any known contraindication to laser therapy

- Allergy or contraindication to acetaminophen

- Previous implant placement or surgical intervention at the same site within the past 6 months

- Current heavy smoking (more than 12 cigarettes per day) or having quit smoking within the past 6 months

Study Procedures

If you agree to participate, the following steps will be undertaken:

1. Preoperative Evaluation: A full clinical examination and cone-beam computed tomography (CBCT) will be performed to evaluate bone volume and suitability for implant placement.

2. Randomization: You will be randomly assigned to one of three groups:

- Group 1: Laser therapy with 650 nm diode laser

- Group 2: Laser therapy with 810 nm diode laser

- Group 3: Control group (no laser therapy)

3. Surgical Procedure: All participants will receive a standardized dental implant using the same implant type (diameter: 3.5–4.0 mm; length: 10 mm) from a single manufacturer.

4. Postoperative Care: Your pain levels will be measured using the Numeric Rating Scale (NRS), and you will be asked to document any analgesic use in a provided log sheet.

5. Follow-Up: You will attend follow-up appointments at 24, 48, and 72 hours post-surgery to assess pain levels and healing progress.

Risks and Discomforts

The potential risks involved in this study are minimal and similar to routine dental implant procedures. These may include:

- Mild to moderate postoperative pain

- Swelling or bruising

- Temporary numbness or tingling

- Rare possibility of infection

Laser therapy may cause a mild warming sensation or tingling at the site of application. No known severe side effects are associated with the low-level laser parameters used in this study.

Benefits

Although there may be no direct personal benefit, your participation will contribute valuable data to ongoing research in laser-assisted dentistry. Indirect benefits may include better pain control and enhanced healing following implant surgery.

Confidentiality

Your identity and all personal data will be kept strictly confidential. All collected information will be anonymized and coded. Data will be used exclusively for scientific purposes, including presentations or publications, without revealing any identifying information.

This study will be registered at ClinicalTrials.gov in compliance with international trial registration requirements.

Voluntary Participation

Participation in this study is entirely voluntary. You have the right to refuse to participate or withdraw your consent at any time, without any consequences for your ongoing or future medical or dental care.

Contact Information

If you have questions about this study or your rights as a participant, you may contact:

Principal Investigator: Dr. Mohammad Yehya Abdussalam

Phone:01140228214

Email: std.yehya80@niles.edu.eg

Institutional Affiliation: NILES, Cairo University, Egypt

Consent Statement

I confirm that I have read and understood the information in this consent form. I have had the opportunity to ask questions, and all of my questions have been answered to my satisfaction. I voluntarily agree to participate in this clinical research study.

Participant's Name: ______________________________

Signature: ______________________________

Date: ______________________________

Investigator's Name: ______________________________

Signature: ______________________________

Date: ______________________________
