# Supplementary material for: Analgesic outcomes of 650 nm versus 810 nm diode laser photobiomodulation after dental implant placement in a randomized controlled trial
Source: Sci Rep. 2026 Apr 21;16:19671. doi: 10.1038/s41598-025-32248-8 (PMC13315321; doi:10.1038/s41598-025-32248-8)
Supplement: Supplementary file 2 — Supplementary Information 2. [file 41598_2025_32248_MOESM2_ESM.pdf]

NRS Pain Score and Analgesic Intake Log Sheet

Participant Name: ______________________

Study ID: ______________________

Date of Implant Surgery: ______________________

Time Point	Clock Time	Pain Score (0–10)	Acetaminophen Taken (Yes/No)	Dosage (mg)	Additional Notes
0 hours (Surgery End Time)					
2 hour					
6 hour					
12 hour					
24 hour					
48 hour					
72 hour					

Instructions for Completing the Pain and Medication Log Sheet

Please follow these simple steps to help us accurately assess your recovery:

Clock Time: The exact time for each entry (based on your surgery end time) will be filled in by the study investigator — **please do not change this column**.

✅ **Your Tasks (to be completed at each time point listed):**

- Please record your pain level (Pain Score) using the Numeric Rating Scale (NRS) at the specified **Clock Time** below.
- Use the scale from 0 to 10, where 0 means "No pain" and 10 means "Worst pain imaginable". Refer to the table below for a detailed description of each pain score.
- The score should reflect the **worst pain since the last scheduled Clock Time**, even if you missed recording it.
- If you forget to fill in a time, **leave it blank** and write the **reason and your best estimate** of the pain in the **Notes** section.
- In the **"Acetaminophen Taken"** column, write **"Yes"** only if you took a 1000 mg dose of acetaminophen within **4 hours before** the listed time, otherwise write **"NO".**
- Use the Notes column to mention any of the following:
    - Missed or delayed a pain score recording (and why)
    - Took another pain medication (name it if possible)
    - Took acetaminophen earlier than advised (explain the reason)
    - Any side effects, unusual symptoms, or important comments

Reminder:

- Please take **1000 mg acetaminophen only if your pain score exceeds 3.**
- Try to stick to the schedule and fill in the log as soon as possible after each listed time point.
- Keep this sheet safe and return it as instructed.
- For any questions or clarifications, please contact principal investigator at **01140228214**.

Numeric Rating Scale (NRS)

Pain Score	Description
**0**	**No pain**
**1-3**	**Mild pain:** Pain is present but does not interfere with daily activities.
**4-6**	**Moderate pain:** Pain interferes with some activities but is manageable.
**7-9**	**Severe pain:** Pain significantly limits activities and requires intervention.
**10**	**Unbearable pain**:Worst possible pain.

Important:

- If you experience any unexpected side effects, please contact us immediately.

Signature of Participant: ________________________

Date: ________________________

Contact for Support

Principal Investigator:

Dr. Mohammad Mahmoud Yehya Abdussalam

National Institute of Laser Enhanced Sciences

Email:std.yehya80@niles.edu.eg

Phone: 01140228214

Thank you for your participation!
