# Supplementary material for: Analgesic outcomes of 650 nm versus 810 nm diode laser photobiomodulation after dental implant placement in a randomized controlled trial
Source: Sci Rep. 2026 Apr 21;16:19671. doi: 10.1038/s41598-025-32248-8 (PMC13315321; doi:10.1038/s41598-025-32248-8)
Supplement: Supplementary file 3 — Supplementary Information 3. [file 41598_2025_32248_MOESM3_ESM.pdf]

**Modified SF-OHIP-14 Questionnaire Form**

***Study Title:***

# Analgesic and OHRQoL Outcomes of 650 nm vs 810 nm Diode Laser Photobiomodulation After Dental Implant Placement: A Randomized Controlled Trial

### *Patient Information*

**Participant ID:** ____________________________

**Date:** ____________________________

**Patient Details:**

- **Name (Optional):** ______________________________________________
- **Sex:** ☐ Male ☐ Female ☐ Other
- **Age:** _________ years
- **Contact Number (Optional):** ___________________________________
- **General Health Status:** ☐ Excellent ☐ Good ☐ Fair ☐ Poor

### *Explanation of Procedure and Purpose*

You are participating in a clinical study that investigates the effects of photobiomodulation (PBM) therapy in reducing pain and improving recovery after dental implant placement. This questionnaire is part of the study's secondary aim, which is to evaluate how the procedure and PBM treatment have impacted your quality of life.

By completing this form, you are helping researchers understand the effectiveness of PBM therapy in improving daily functions, managing discomfort, and enhancing overall well-being after dental implant placement. This is part of the study you have already consented to, and your responses will remain confidential.

### *Instructions for Completing the Form*

- Please answer all questions honestly, based on your experiences over the past week.
- For each question, mark the option that best represents how often you experienced the described issue:
  - **0:** Never
  - **1:** Hardly Ever
  - **2:** Occasionally
  - **3:** Fairly Often
  - **4:** Very Often

## Quality of Life Questionnaire

### *Functional Limitation*

1. Have you had difficulty speaking clearly due to pain or discomfort in your mouth following the dental implant procedure?

☐ 0 ☐ 1 ☐ 2 ☐ 3 ☐ 4

2. Have you noticed any changes in your sense of taste or chewing due to discomfort in your mouth or dental implants?

☐ 0 ☐ 1 ☐ 2 ☐ 3 ☐ 4

### *Physical Pain*

3. Have you experienced painful aching in your mouth after the dental implant procedure?
   ☐ 0 ☐ 1 ☐ 2 ☐ 3 ☐ 4
4. Have you found it uncomfortable to eat certain foods due to pain or sensitivity after the dental implant placement?
   ☐ 0 ☐ 1 ☐ 2 ☐ 3 ☐ 4

### *Psychological Discomfort*

5. Have you felt self-conscious because of discomfort or the appearance of your dental implant area?
   ☐ 0 ☐ 1 ☐ 2 ☐ 3 ☐ 4

6. Have you felt tense or stressed because of pain or sensitivity in your mouth following the implant procedure?
   ☐ 0 ☐ 1 ☐ 2 ☐ 3 ☐ 4

### *Physical Disability*

7. Has your diet been unsatisfactory due to pain or discomfort from your dental implant site?
   ☐ 0 ☐ 1 ☐ 2 ☐ 3 ☐ 4
8. Have you had to interrupt meals because of discomfort or pain in your mouth?
   ☐ 0 ☐ 1 ☐ 2 ☐ 3 ☐ 4

### *Psychological Disability*

9. Have you found it difficult to relax because of discomfort or pain in your mouth?
   ☐ 0 ☐ 1 ☐ 2 ☐ 3 ☐ 4
10. Have you felt embarrassed due to the condition of your mouth or the dental implant procedure?
    ☐ 0 ☐ 1 ☐ 2 ☐ 3 ☐ 4

### *Social Disability*

11. Have you been irritable with others because of pain or discomfort related to your dental implant?
    ☐ 0 ☐ 1 ☐ 2 ☐ 3 ☐ 4
12. Have you experienced difficulty completing your usual daily tasks due to discomfort or pain in your mouth?
    ☐ 0 ☐ 1 ☐ 2 ☐ 3 ☐ 4

### *Handicap*

13. Have you felt that life in general was less satisfying because of pain or discomfort following the dental implant procedure?
    ☐ 0 ☐ 1 ☐ 2 ☐ 3 ☐ 4
14. Have you been completely unable to function due to pain or discomfort in your mouth?
    ☐ 0 ☐ 1 ☐ 2 ☐ 3 ☐ 4

### *Acknowledgment and Consent*

By completing this form, I confirm that I understand its purpose and have answered the questions truthfully to the best of my ability.

**Signature of Participant:** ___________________________

**Date:** ___________________________

## For Research Use Only

**Reviewed by:** _____________________________

**Date of Review:** _____________________________
